# Gradual stabilization and narrowing of bone tunnels following primary anterior cruciate ligament reconstruction

**DOI:** 10.1002/ksa.12398

**Published:** 2024-08-02

**Authors:** Di Liu, Wenhao Lu, Djandan Tadum Arthur Vithran, Qing Bi, Zheping Hong, Xu Liu, Dongliang Yuan, Can Chen, Wenfeng Xiao, Yusheng Li

**Affiliations:** ^1^ Department of Orthopedics, Xiangya Hospital Central South University Changsha China; ^2^ Department of Orthopedic Surgery, Peking Union Medical College Hospital Peking Union Medical College and Chinese Academy of Medical Sciences Beijing China; ^3^ National Clinical Research Center for Geriatric Disorders, Xiangya Hospital Central South University Changsha China; ^4^ Department of Sports Medicine Zhejiang Provincial People's Hospital and People's Hospital of Hangzhou Medical College Hangzhou Zhejiang China

**Keywords:** anterior cruciate ligament reconstruction, clinical outcomes, computed tomography, graft maturity, tunnel widening

## Abstract

**Purpose:**

The purpose of this study is to dynamically assess variations in tunnel diameters following anterior cruciate ligament reconstruction (ACLR) and investigate correlations with patient‐reported outcomes (PROs) and graft maturity based on signal‐to‐noise quotient (SNQ).

**Methods:**

Tunnel diameter and tunnel position were measured using three‐dimensional models derived from computed tomography (CT) data. Postoperative graft maturity and integration were evaluated using magnetic resonance imaging (MRI). Clinical outcomes were assessed through PROs, which included the International Knee Documentation Committee Subjective Knee Evaluation Form, Knee Injury and Osteoarthritis Outcome Scores and Lysholm scores. The correlation between tunnel enlargement extent, PROs and SNQ values, as well as correlations between confounding factors, tunnel diameter differences and SNQ were analyzed.

**Results:**

A total of 73 participants underwent primary ACLR and scheduled follow‐ups. At the segment of the articular aperture, the femoral tunnel was enlarged by 32.3% to 10.4 ± 1.6 mm (*p* < 0.05), and the tibial tunnel was widened by 17.2% to 9.6 ± 1.2 mm (*p* < 0.05) at the 6‐month follow‐up. At 1 year postoperatively, diameters at the articular aperture were not further increased on the femoral (n.s.) and tibial (n.s.) sides. In early postoperative follow‐up, the femoral tunnel was anteriorly and distally shifted, coupled with posterior and lateral deviation involving the tibial side, exhibiting minimal migration at 1‐year follow‐up. The degree of tunnel widening was not correlated with PROs and SNQ values. Age, gender, body mass index (BMI), time from surgery to follow‐up, concomitant injuries and autograft type were not correlated with tunnel diameter differences and SNQ.

**Conclusions:**

The femoral and tibial bone tunnels exhibited eccentrical widening and gradually stabilized at 1 year following ACLR. Furthermore, the enlarged bone tunnels were not correlated with unsatisfied PROs and inferior graft maturity.

**Level of Evidence:**

Level IV.

**Abbreviations**
2D: two‐dimensional
3D: three‐dimensional
ACL: anterior cruciate ligament
ACLR: anterior cruciate ligament reconstruction
AL: anterolateral
AM: anteromedial
BMI: body mass index
CT: computed tomography
FoV: field of view
fs PD: fat‐saturated proton density
ICC: intra‐class correlation coefficient
IKDC: The International Knee Documentation Committee
KOOS: Knee Injury and Osteoarthritis Outcome Scores
MRI: magnetic resonance imaging
OA: osteoarthritis
PCL: posterior cruciate ligament
PRO: patient‐reported outcome
QoL: quality of life
ROI: region of interest
ROM: range of motion
SNQ: signal‐to‐noise quotient

## INTRODUCTION

Over the past decade, anterior cruciate ligament (ACL) reconstruction (ACLR) has been regarded as the predominant treatment modality to restore knee stability and functional activities, especially for young or athletic patients with ACL rupture [7, 8]. However, the considerable increase in surgical reconstructions is concomitant with widely reported tunnel widening on both femoral and tibial sides [3, 11, 30, 35, 40]. There is disagreement concerning the adverse effects of larger bone tunnels on clinical outcomes. Widened bone tunnels theoretically cause anterior tibial laxity, knee instability, graft failure and revision ACLR [47]. However, several studies have demonstrated that tunnel widening does not correlate with inferior clinical results following primary and revisionary surgeries [6, 41, 44, 49].

The accurate evaluation of tunnel diameters plays a fundamental role in aetiological and morphological studies concerning the potential impact of enlarged bone tunnels on clinical outcomes. Plain radiography, computed tomography (CT) and magnetic resonance imaging (MRI) have been conventionally adopted to measure the postoperative diameters of bone tunnels [29, 39, 41]. However, these two‐dimensional (2D) methods have several disadvantages that make it impossible to accurately measure tunnel diameters [11, 34, 35]. Notably, three‐dimensional (3D) models provided a promising strategy in which bone tunnel diameters can be accurately measured under direct vision [13, 32, 38].

This study sought to depict the diameter variation in bone tunnels following ACLR by constructing 3D models and by investigating the correlation of the degree of tunnel widening with clinical outcomes using patient‐reported outcomes (PROs) and graft maturity using signal‐to‐noise quotient (SNQ). It was hypothesized that bone tunnels would not be constantly widened, and the extent of enlargement would not affect clinical outcomes and graft healing.

## MATERIALS AND METHODS

The Institutional Review Board of Xiangya Hospital (No. 202201039) approved the protocol for this study, and written informed consent was obtained from all participants.

This was a single‐centre observational study. All participants were prospectively recruited from March 2022 to February 2023. Eligibility criteria included unilateral ACL, primary ACLR and being aged 14–50. All participants received follow‐ups at 6 months and 1 year postoperatively. Patients with multi‐ligamentous injury, re‐rupture of autografts, postoperative infection, history of knee joint operations, osteoarthritis (OA), or contraindications for CT or MRI were excluded.

### Surgical techniques

All patients underwent standard ACLR using the ‘all‐inside’ technique [9], which was performed by the same orthopaedic surgeon. The semitendinosus and gracilis tendons harvested were initially duplicated by threading through an adjustable‐loop suspension device (TightRope button, Arthrex), which were further quadrupled. The loose ends of the graft were then joined together using a high‐strength multi‐strand suture (FiberWire No.0, Arthrex). Subsequently, the prepared graft was pre‐tensioned to a final length not exceeding 70 mm. Concurrently, the ligamentous tear, meniscal injury, or cartilage lesions were checked during arthroscopy. Tunnel drilling was conducted to create a 25‐mm femoral socket and a 30‐mm tibial socket at the locations corresponding to the ACL footprints. Then, the autograft was threaded through all of the tunnels and securely fastened using the TightRope button (Arthrex). Finally, the reconstructed autograft was tensioned with the full extension of the knee joint.

### Postoperative rehabilitation

All participants were directed to resume their daily lives, including sporting activities, and to adhere to a standard rehabilitation plan. From day 1, patients were encouraged to regain muscle strength and lower extremity range of motion (ROM). In general, the reconstructed knee could achieve flexion of 90° within 2 weeks and attain full ROM within 4 weeks postoperatively. Tolerable weight‐bearing was encouraged as early as possible, and wearing an external protection brace was permitted for 3 months postoperatively. At 6 months after ACLR, patients were allowed to participate in jogging and swimming and to gradually return to all kinds of sports activities 1 year postoperatively. As appropriate, rehabilitation plans were readjusted according to the individual patient's physical condition and surgical treatment.

### Clinical outcome assessment

PROs were utilized to evaluate knee function, pain, activity levels and quality of life (QoL) preoperatively and 6 months and 1 year after ACLR. The International Knee Documentation Committee (IKDC) Subjective Knee score, Knee Injury and Osteoarthritis Outcome Scores (KOOS) and Lysholm scores were included and evaluated in this study.

### Imaging Assessment

**Figure 1:**
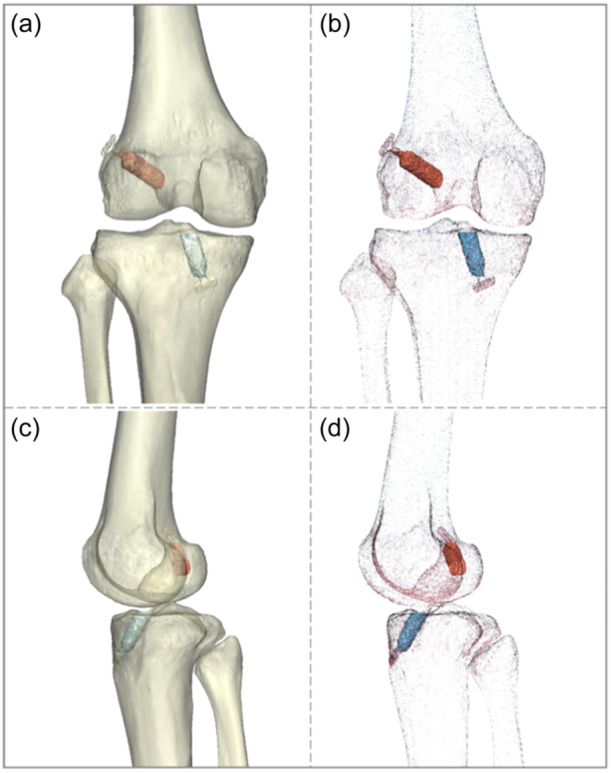
Reconstructed 3D models of the femur, tibia, fibula and bone tunnels under anterior‐posterior view (a, b) and medial‐lateral view (c, d).

To evaluate tunnel widening and shifting, acquired conventional CT data were reconstructed into 3D bone tunnels to measure tunnel diameters and relative positions directly on the first day, 6 months and 1 year postoperatively (Figure 1). All participants were scanned by a CT (Somatom Drive, Siemens Healthcare) on the first postoperative day and at 6‐month and 1‐year follow‐ups. A standard imaging protocol was utilized employing the following parameters: 80 kV and Sn140 kV, 210 mAs and 105 mAs, 1.0‐mm slice thickness, 0.7‐mm slice increment, 0.5‐s rotation time, 32 × 0.6‐mm^2^ detector collimation, 0.7 helical pitch, 512 × 512 matrix size and 250‐mm field of view (FoV). Protective lead clothing was employed to shield the vital glands and organs of the participants.

**Figure 2:**
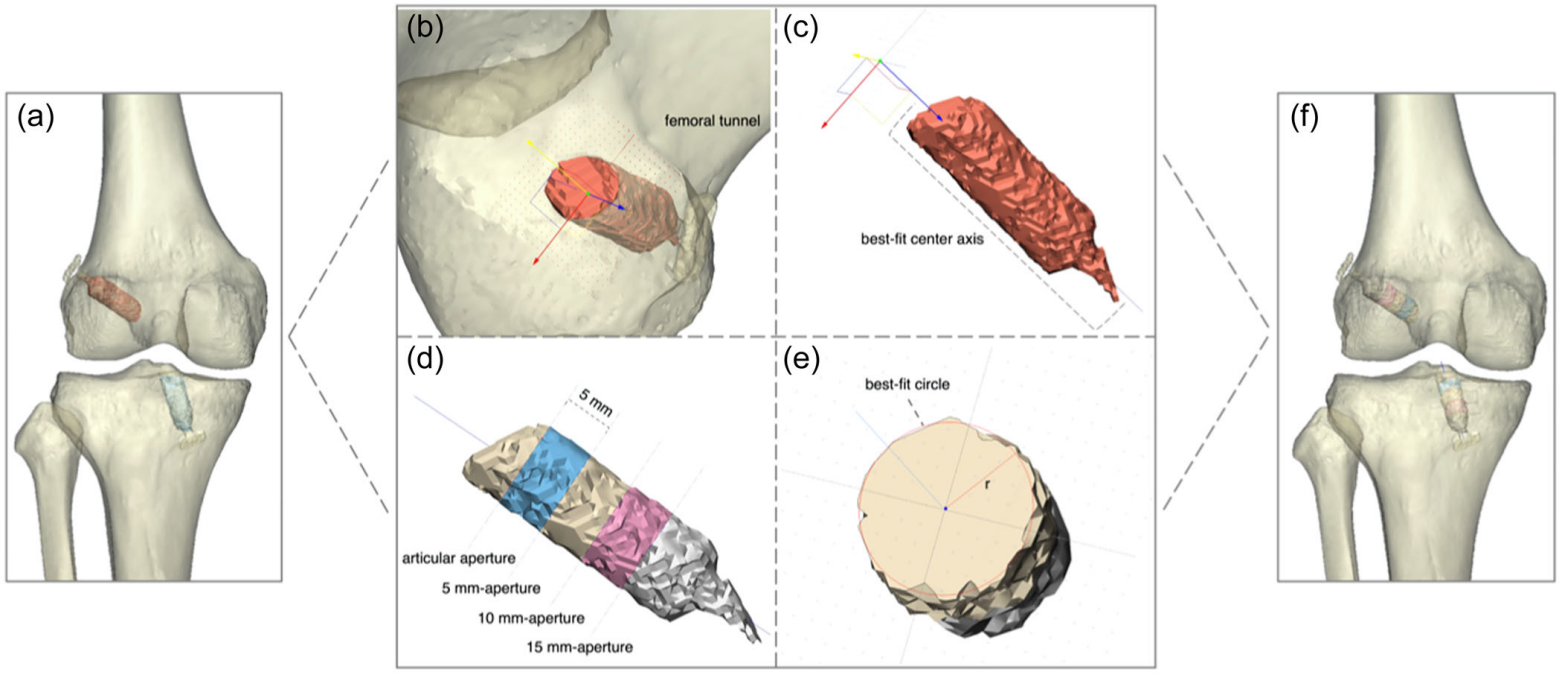
Measurement of tunnel diameters. (a) and (f) Anterior view of a 3D model of a right knee. (b) 3D models of the femur (ash grey) and the femoral tunnel (red). (c) A best‐fit axis was set to the geometric centre of the femoral tunnel. (d) The femoral tunnel was segmented into four measurement levels with a 5‐mm interval (aperture, 5‐mm aperture, 10‐mm aperture and 15‐mm aperture). (e) Each segment had a best‐fit circle, and the corresponding diameter was regarded as the primary outcome.

DICOM raw data were exported to image post‐processing software Materialise Mimics, v.21.0. The femurs, tibias and bone tunnels were preliminarily segmented by algorithms based on density variations between bone and soft tissues on CT images. Then, a manual revision was conducted to ensure the bone tunnels were accurately outlined and appeared as separate segments from femurs or tibias. The bone tunnel models were then imported into 3D modeling software (Materialise 3‐Matic, v.13.0), in which the analytical 3D bone tunnels were created. In this step, a centre axis, as the automated centre line, was fitted into the whole tunnel length with direct visualization. Along this best‐fit centre axis, circles were fitted to the surface of tunnel walls with 5‐mm intervals from the articular aperture to the cortical bone. In this study, four apertures were identified: an articular aperture, 5 mm from the aperture, 10 mm from the aperture and 15 mm from the aperture. The diameters of these best‐fit circles were then used to measure tunnel diameters (Figure 2).

**Figure 3:**
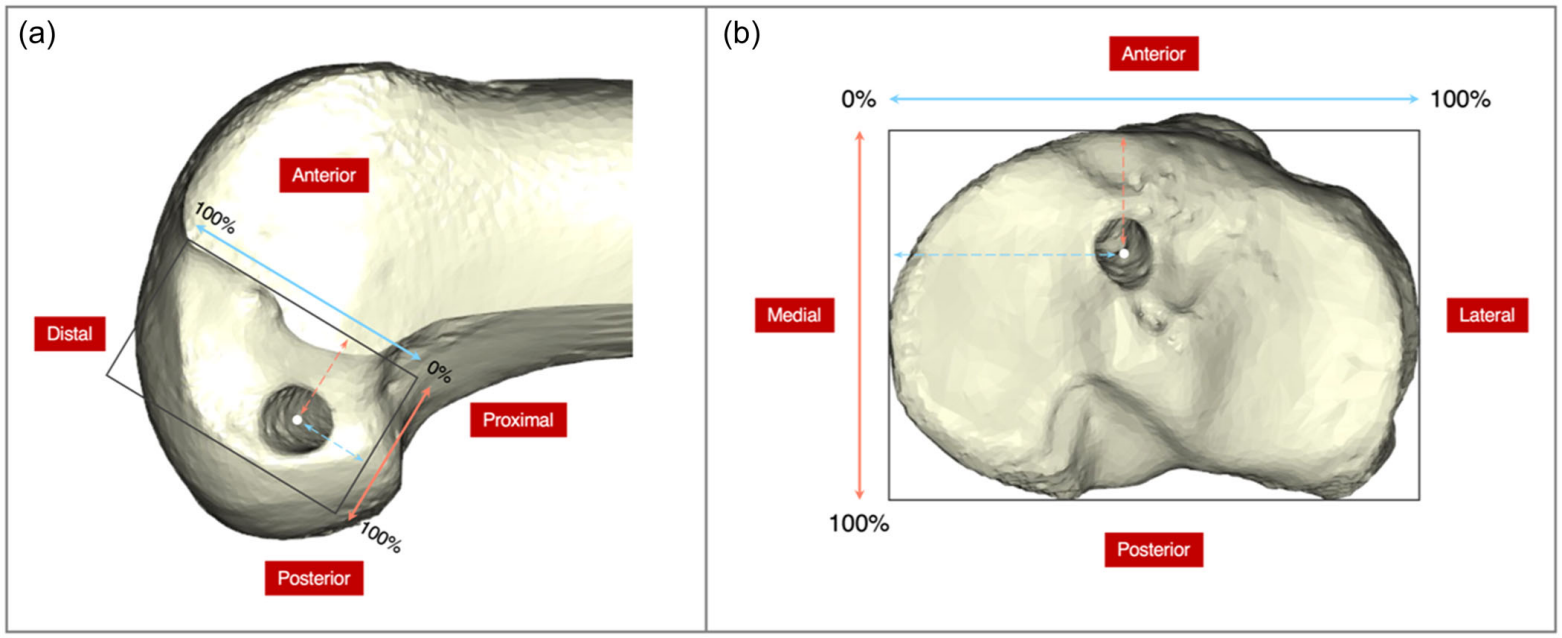
Measurement of tunnel positions. (a) Femoral tunnel position: The anterior–posterior position was described as a percentage: length of dashed orange–red line/length of solid red line * 100%. The distal–proximal position was calculated as a percentage: length of dashed blue line/length of solid blue line * 100%. (b) Tibial tunnel position: The anterior–posterior position was described as a percentage: length of dashed orange–red line/length of solid red line * 100%. The medial–lateral position was calculated as a percentage: length of dashed blue line/length of solid blue line * 100%.

The tunnel position and its shifting were evaluated using 3D models as well by engaging the quadrant method [25, 26, 32]. In this study, the 3D femur was horizontally placed in a strictly lateral orientation, ensuring an overlap of lateral and medial femoral condyles. Then, the femoral model was oriented in a neutral position to address the medial femoral condyle through the highest point of the intercondylar notch. Finally, the femur underwent rotation to achieve the strictly lateral position. Similarly, the tibial condyle was positioned horizontally parallel in the posterior view. A 90° rotation was then applied to present the top view of the tibial plateau to ensure that the posterior articular margins of the medial and lateral condyles were aligned. The position change of the central tunnel aperture was regarded as the direction of tunnel shifting. The medial‐lateral perspective of the lateral femoral condyle and the top view of the tibial plateau were employed to assess the relative position of the tunnel aperture by the quadrant method [4, 17, 25, 31]. Rectangular reference frames were delineated using the border of the intercondylar notch or proximal tibia to establish femoral and tibial tunnel positions, respectively (Figure 3).

Model reconstruction and manual adjustment were executed by an orthopaedic investigator who maintained independence from surgical procedures and patient care.

### MRI‐based assessment of graft maturity

**Figure 4:**
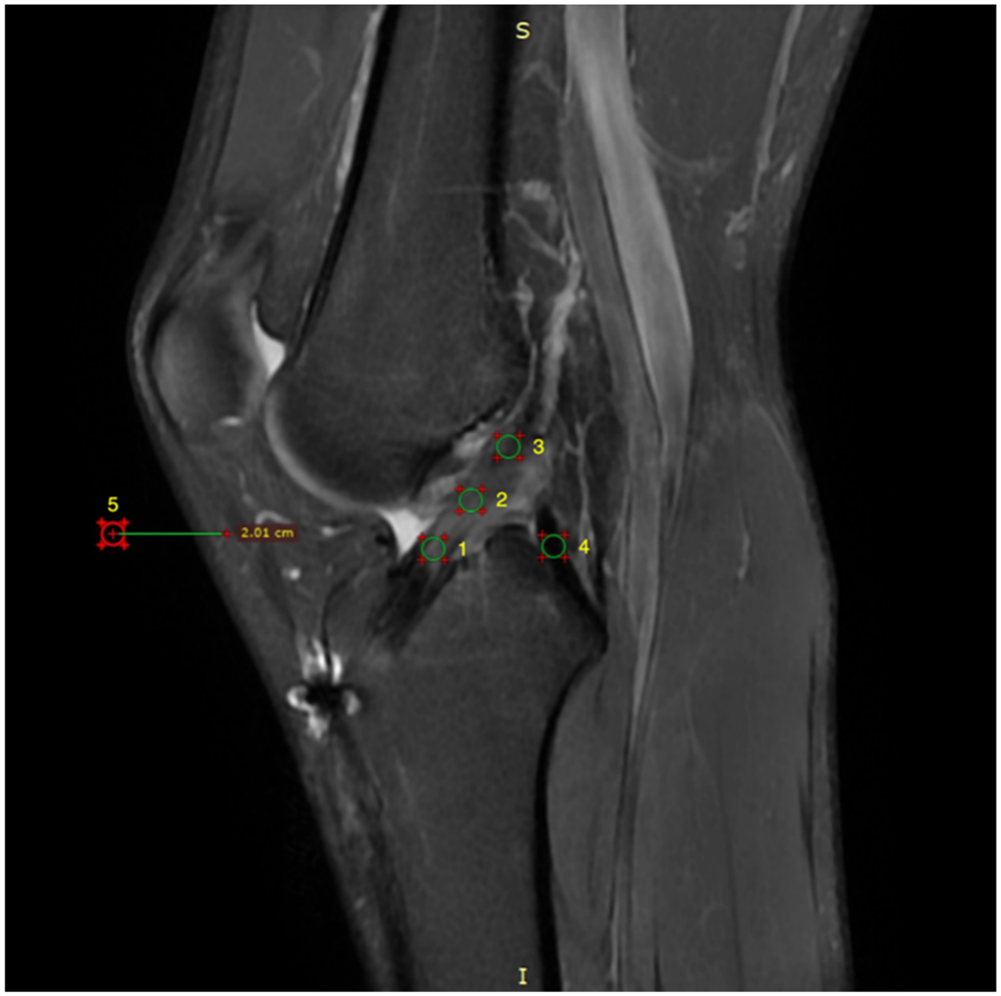
Calculation of graft SNQ. Three circular ROIs were evenly positioned along a line on the distal, mid‐substance and proximal regions of the autograft, and one circular ROI was placed at the base of the PCL, central to its broad tibial attachment. The background site was chosen approximately 2 cm anterior to the quadriceps tendon. ROI, region of interest; SNQ, signal/noise quotient.

All reconstructed knees underwent MRI imaging at 1‐year follow‐up to evaluate graft maturity. Oblique sagittal images obtained from fat‐saturated proton density (fs PD) sequences were chosen for measurement of autograft SNQ values using a RadiAnt Medical Image Viewer (v.2021.02, Medixant). A total of five 12.4‐mm² circular regions of interest (ROIs) were chosen, including the distal, median and proximal parts of the intra‐articular autografts, posterior cruciate ligament (PCL) and background. SNQ was calculated as follows: SNQ = (SNQ_ACL − SNQ_PCL)/SNQ_Background (Figure 4).

### Measurement reliability

Two investigators independently and repeatedly measured tunnel diameters, tunnel positions and SNQ values with a 1‐month interval. The intra‐ and inter‐observer reliability for radiographic and 3D measurements was good to excellent (Table 1).

**Table 1:** Intra‐ and inter‐reliability on the measurements of tunnel diameters, tunnel positions and SNQ.

	Tunnel diameter		Tunnel position		SNQ	
	ICC	95% CI	ICC	95% CI	ICC	95% CI
Intra‐observer						
Observer 1	0.91	0.86–0.95	0.93	0.87–0.97	0.85	0.79–0.89
Observer 2	0.93	0.87–0.97	0.87	0.81–0.92	0.86	0.80–0.90
Inter‐observer	0.89	0.83–0.93	0.90	0.84–0.94	0.81	0.75–0.85

Abbreviation: ICC, intra‐class correlation coefficient; SNQ, signal‐to‐noise quotient; 95% CI, 95% confidence interval.

### Statistical analysis

Descriptive statistics accurately describe participant demographic characteristics. A comparison of postoperative 3D and intraoperative bone tunnel diameters was carried out using the Mann–Whitney *U* test. Wilcoxon's signed‐rank test for paired samples or paired Student's *t* test was used to assess and compare tunnel diameters, tunnel positions and clinical outcomes. Kruskal–Wallis *H* test testing was employed to compare SNQ values across various parts of autografts. Analyses using Pearson's or Spearman's rank correlations explored the relationships between tunnel diameters, SNQ values and PROs. Multiple regression analysis was performed to identify which of the confounding factors were correlated with differences in tunnel diameters ab initio to 1‐year follow‐up and SNQ values of full autografts at 1‐year follow‐up. The independent factors were age, gender, body mass index (BMI), time from surgery to follow‐up, meniscal injury, chondral injury and autograft type. Intra‐class correlation coefficients (ICCs) were calculated to assess both intra‐ and inter‐observer agreements for measurements of imaging findings utilizing a two‐way random model. Statistical analyses were performed using SPSS software (v.26, IBM Corp.), and the threshold for statistical significance was set at *p* < 0.05. Based on the means and standard deviations of paired differences of tunnel diameters from Monaco et al. [38], a sample size of 22 achieved a power of 92% with a significance level of 0.02 and a dropout rate of 20%. Sample size calculation for paired means using Wilcoxon tests was performed by PASS software (v.15.0.5, NCSS).

## RESULTS

### Primary participant demographics

A total of 73 participants (47 males and 26 females) were enroled and followed up in this study. The average age at ACLR was 26.5 ± 8.6 years. The median duration of follow‐up was 374 days (range 345–452 days). The main participant demographics are shown in Table 2.

**Table 2:** Main demographics of participants.

Age, years^a^	26.5 ± 8.6 (14–47)
Sex, male/female	47/26
BMI, kg/m^2^ a	24.2 ± 4.0 (16.9–32.9)
Time from injury to surgery, months^b^	1.00 (0.1–121.7)
Time from surgery to final follow‐up, days^b^	374 (345–452)
Torn side of ACL, left/right	38/35
Meniscal injury, *n* (%)	52 (71.2%)
Chondral injury, *n* (%)	26 (35.6%)
Autograft type, ST/ST+G	48/25
Drill diameter, mm^b^	
Femoral tunnel	8.0 (7.0–9.0)
Tibial tunnel	8.0 (7.0–9.0)

Abbreviations: ACL, anterior cruciate ligament; BMI, body mass index; G, gracilis; n, number; ST, semitendinosus.

a Data are expressed as mean ± SD (range values).

^b^ Data are expressed as median (range values).

### Patient‐reported clinical outcomes

All postoperative PROs indicated a significant improvement compared with preoperative evaluations (*p* < 0.05) (Table 3). Participants returned to various sports activities, and none of the revision surgeries, or suffered joint infection, joint instability, or other complications occurred during the follow‐up period.

**Table 3:** Patient‐reported clinical outcomes.

	Pre‐op	6 M post‐op	1 Y post‐op	*p* Value		
				Δ PROs _6 M–Pre_	Δ PROs _1 Y–Pre_	Δ PROs _1 Y–6 M_
IKDC	47.4 ± 21.8^a^	79.3 (73.6–83.9)^b^	89.2 ± 5.4^a^	**<0.001** **	**<0.001** *	**<0.001** **
Lysholm	54.0 (36.0–69.0)^b^	86.0 (69.0–94.0)^b^	90.0 (85.0–95.0)^b^	**<0.001** **	**<0.001** **	**<0.001** **
KOOS‐symptoms	57.6 ± 24.1^a^	85.7 (75.0–92.9)^b^	89.3 (82.1–96.4)^b^	**<0.001** **	**<0.001** **	**<0.001** **
KOOS‐pain	66.7 (44.4–83.3)^b^	88.9 (83.3–97.2)^b^	94.4 (91.7–100.0)^b^	**<0.001** **	**<0.001** **	**<0.001** **
KOOS‐ADL	75.0 (38.2–88.2)^b^	95.6 (88.2–98.5)^b^	97.1 (94.1–98.5)^b^	**<0.001** **	**<0.001** **	**<0.001** **
KOOS‐sport	44.8 ± 27.0^a^	75.0 (65.0–85.0)^b^	80.0 (70.0–90.0)^b^	**<0.001** **	**<0.001** **	**<0.001** **
KOOS‐QoL	45.1 ± 24.6^a^	75.0 (56.3 −87.5)^b^	87.5 (75.0–93.8)^b^	**<0.001** **	**<0.001** **	**<0.001** **

*Note*: Δ Indicates the PROs difference among the first day, 6 months and 1 year postoperatively.

Abbreviations: ADLs, activities of daily living subscale; IKDC, International Knee Documentation Committee; KOOS, Knee Injury and Osteoarthritis Outcome Scores; pre‐op, preoperatively; PRO, patient‐reported outcome; QoL, quality of life subscale; Sport, sport and recreation subscale; 6 M post‐op, 6 months postoperatively; 1 Y post‐op, 1 year postoperatively.

a Data are expressed as mean ± SD.

^b^ Data are expressed as median (*P*_25_–*P*_75_).

* A *p* value of <0.05 indicates statistical significance with the paired *t* test and is highlighted in bold.

** A *p* value of <0.05 indicates statistical significance with the Wilcoxon signed‐rank test and is highlighted in bold.

### Postoperative tunnel diameters

**Figure 5:**
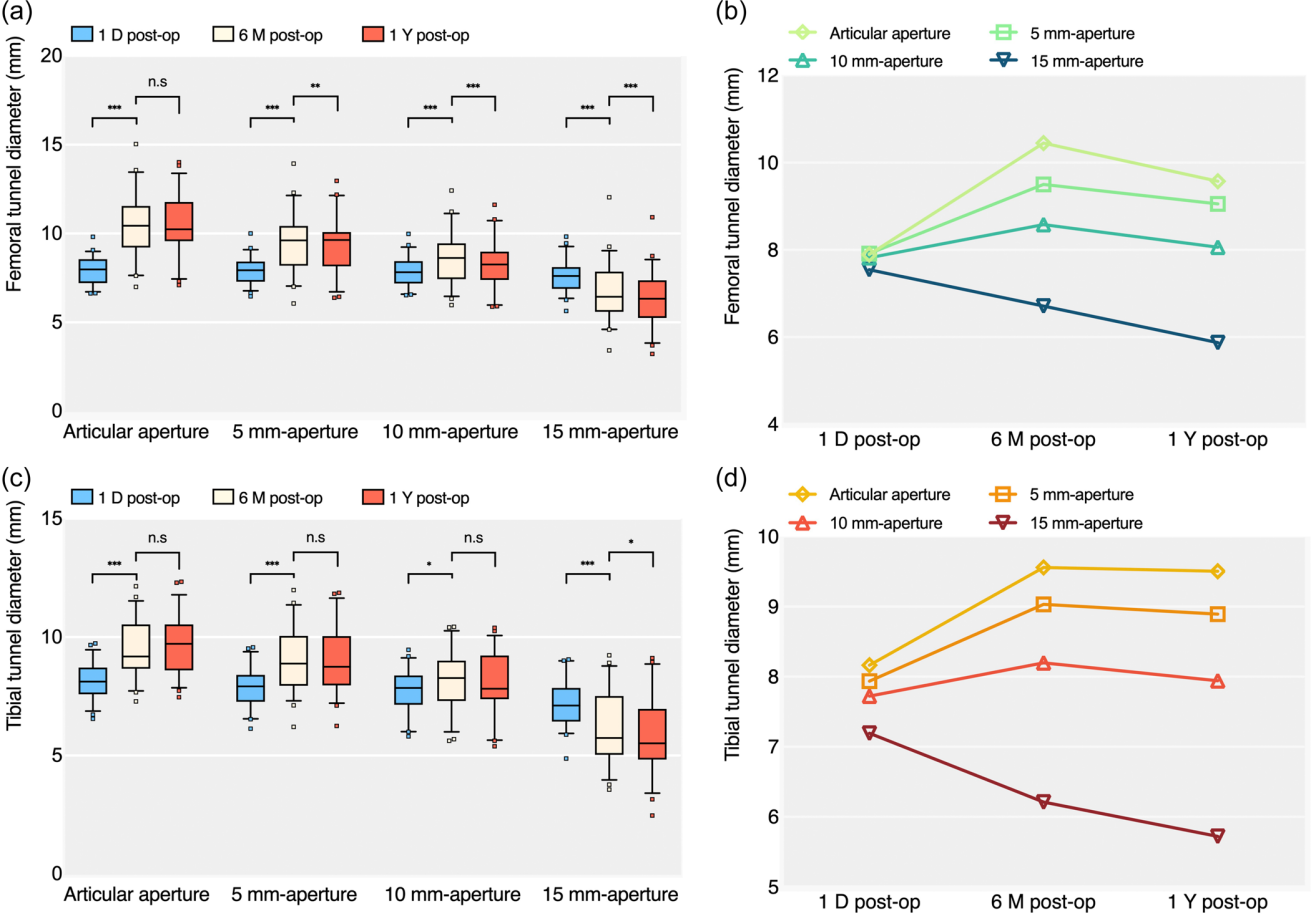
Boxplot graphs (a, c) and line graphs (b, d) demonstrating variations and changes in femoral and tibial tunnel diameters after primary ACLR at various follow‐up time points and across different tunnel segments. ACLR, anterior cruciate ligament reconstruction; 1 D post‐op, 1st day postoperatively; 6 M post‐op, 6 months postoperatively; 1 Y post‐op, 1 year postoperatively.

Median drill diameters were 8.0 mm (range 7.0–9.0 mm) for both femoral and tibial tunnels. There was no significant difference in tunnel diameters between first‐day 3D models and intraoperative bone tunnels on the femoral (7.9 ± 0.8 mm vs. 8.0 [7.0–9.0] mm, n.s.) and tibial sides (8.2 ± 0.7 mm vs. 8.0 [7.0–9.0] mm, n.s.). At the segment of the tunnel aperture, the diameter of the femoral side was increased by 32.3% to 10.4 ± 1.6 mm at the 6‐month follow‐up (*P* < 0.05); on the tibial side, the diameter of the tunnel aperture was enlarged by 17.2% to 9.6 ± 1.2 mm (*P* < 0.05) Furthermore, there was no evidence of a difference between the 6‐month and 1‐year tunnel diameters on femoral (n.s.) and tibial (n.s.) sides (Table 4 and Figure 5).

**Table 4:** Tunnel diameter on the first day, 6 months and 1 year after ACLR.

	Tunnel diameter (mm)			Δ Tunnel diameter _6 M–1 D_		Δ Tunnel diameter _1 Y–1 D_		Δ Tunnel diameter _1Y–6 M_	
	1 D post‐op	6 M post‐op	1 Y post‐op	Mean ± SD	*p* Value	Mean ± SD	*p* Value	Mean ± SD	*p* Value
Femoral tunnel									
Articular aperture	7.9 ± 0.8	10.5 ± 1.6	10.5 ± 1.6	2.6 ± 1.3	**<0.001**	2.6 ± 1.2	**<0.001**	0.0 ± 0.5	n.s.
5 mm from the aperture	7.9 ± 0.7	9.5 ± 1.6	9.3 ± 1.5	1.6 ± 1.2	**<0.001**	1.4 ± 1.1	**<0.001**	−0.2 ± 0.5	**<0.01**
10 mm from the aperture	7.8 ± 0.8	8.6 ± 1.4	8.3 ± 1.3	0.8 ± 1.1	**<0.001**	0.5 ± 1.1	**<0.01**	−0.3 ± 0.4	**<0.001**
15 mm from the aperture	7.5 ± 0.9	6.7 ± 1.6	6.3 ± 1.5	−0.8 ± 1.4	**<0.001**	−1.2 ± 1.3	**<0.001**	−0.4 ± 0.6	**<0.001**
Tibial tunnel									
Articular aperture	8.2 ± 0.7	9.6 ± 1.2	9.6 ± 1.2	1.4 ± 0.9	**<0.001**	1.4 ± 0.9	**<0.001**	0.0 ± 0.4	n.s.
5 mm from the aperture	7.9 ± 0.8	9.0 ± 1.3	9.1 ± 1.3	1.1 ± 1.0	**<0.001**	1.1 ± 1.1	**<0.001**	0.0 ± 0.4	n.s.
10 mm from the aperture	7.7 ± 0.8	8.2 ± 1.2	8.1 ± 1.3	0.5 ± 1.1	**<0.05**	0.2 ± 1.2	**<0.05**	−0.1 ± 0.5	n.s.
15 mm from the aperture	7.2 ± 0.9	6.2 ± 1.5	5.9 ± 1.5	−1.0 ± 1.5	**<0.001**	−1.3 ± 1.5	**<0.001**	−0.3 ± 0.8	**<0.05**

*Note*: Data are expressed as mean ± SD. A *p* value of <0.05 indicates statistical significance followed by Bonferroni correction and is highlighted in bold. Δ Indicates the tunnel diameter difference among the first day, 6 months and 1 year postoperatively.

Abbreviations: ACLR, anterior cruciate ligament reconstruction; 1 D post‐op, 1st day postoperatively; 6 M post‐op, 6 months postoperatively; 1 Y post‐op, 1 year postoperatively.

### Postoperative tunnel positions

At the 6‐month follow‐up after surgery, the femoral tunnel migrated into the anterior (*p* < 0.05) and distal direction (*p* < 0.05); the tibial tunnel shifted posteriorly (*p* < 0.05) and laterally (*p* < 0.05). Furthermore, at the 1‐year follow‐up, there was a slight anterior and distal migration on the femoral side (*p* < 0.05), and the tibial tunnel exhibited no significant anterior shifting (n.s.) or variation in the medial–lateral direction (n.s.) (Table 5).

**Table 5:** Tunnel position in the first day, 6 months and 1 year after ACLR.

	Tunnel position (mean ± SD, %)			Tunnel position _6 M–1 D_		Tunnel position _1 Y–1 D_		Tunnel position _1Y–6 M_	
	1 D post‐op	6 M post‐op	1 Y post‐op	Mean ± SD	*p* Value	Mean ± SD	*p* Value	Mean ± SD	*p* Value
Femoral tunnel									
Anterior–posterior (%)	54.7 ± 7.0	51.9 ± 7.3	50.8 ± 6.2	−2.9 ± 4.1	**<0.001** *	−3.9 ± 4.2	**<0.001** *	−1.1 ± 3.5	**<0.05** *
Proximal–distal (%)	29.5 ± 5.8	32.7 ± 6.0	33.7 ± 6.2	3.2 ± 2.6	**<0.001** **	4.1 ± 3.0	**<0.001** **	0.9 ± 1.9	**<0.01** **
Tibial tunnel									
Anterior–posterior (%)	36.8 ± 3.7	37.6 ± 3.4	37.5 ± 3.8	0.8 ± 1.7	**<0.01** **	0.7 ± 2.1	**<0.05** **	−0.1 ± 1.7	n.s.*
Medial–lateral (%)	47.2 ± 2.4	48.1 ± 2.1	47.8 ± 1.9	0.8 ± 1.3	**<0.001** *	0.8 ± 1.3	**<0.001** *	−0.1 ± 0.9	n.s.*

*Note*: Data are expressed as mean ± SD. Δ Indicates the tunnel shifting among the first day, 6 months and 1 year postoperatively.

Abbreviations: ACLR, anterior cruciate ligament reconstruction; 1 D post‐op, 1st day postoperatively; 6 M post‐op, 6 months postoperatively; 1 Y post‐op, 1 year postoperatively.

* A *p* value of <0.05 indicates statistical significance with the paired *t* test and is highlighted in bold.

** A *p* value of <0.05 indicates statistical significance with the Wilcoxon signed‐rank test and is highlighted in bold.

### Postoperative graft maturity

SNQ values for the distal of the ACL were minimal compared with the midsubstance and proximal SNQ values (*p* < 0.05) (Table 6).

**Table 6:** Correlation of tunnel diameter difference (Δ) with clinical outcome difference and graft maturity.

	Δ Femoral diameter at aperture_1 Y–1 D_		Δ Tibial diameter at aperture_1 Y–1 D_	
	*r*	*p* Value	*r*	*p* Value
Δ PROs_1 Y–Pre_				
IKDC	0.02^a^	n.s.	0.22^a^	n.s.
Lysholm	0.11^a^	n.s.	0.31^a^	**<0.05**
KOOS‐symptoms	0.03^a^	n.s.	0.20^a^	n.s.
KOOS‐pain	0.01^b^	n.s.	0.11^b^	n.s.
KOOS‐ADL	−0.08^b^	n.s.	0.21^b^	n.s.
KOOS‐sport	0.06^a^	n.s.	0.26^a^	n.s.
KOOS‐QoL	−0.01^b^	n.s.	0.07^b^	n.s.
SNQ				
Distal	−0.03^b^	n.s.	0.34^b^	**<0.05**
Middle	−0.37^b^	**<0.05**	−0.15^b^	n.s.
Proximal	−0.15^b^	n.s.	−0.01^b^	n.s.
Full	−0.28^b^	**<0.05**	0.09^b^	n.s.

*Note*: A *p* value of <0.05 indicates a significant statistical correlation and is highlighted in bold. Δ indicates the differences between tunnel diameter and PROs.

Abbreviations: ADL, activities of daily living subscale; IKDC, International Knee Documentation Committee; KOOS, Knee Injury and Osteoarthritis Outcome Scores; PRO, patient‐reported outcome; QoL, quality of life subscale; SNQ, signal‐to‐noise quotient; Sport, sport and recreation subscale; 1 D post‐op, 1st day postoperatively; 1 Y post‐op, 1 year postoperatively.

^a^ Data are analyzed by the Pearson correlation.

^b^ Data are analyzed by the Spearman rank correlation.

### Comparison of PROs, tunnel diameters and SNQ between participants with <8‐mm and ≥8‐mm graft diameter

PROs, differences in femoral and tibial tunnel diameters, and SNQ values were comparable between groups (graft diameter <8 mm vs. ≥8 mm, n.s.) (Table 7).

**Table 7:** Comparison of PROs, tunnel diameter and SNQ between participants with <8‐mm and ≥8‐mm graft diameter.

	<8‐mm autografts	≥8‐mm autografts	*p* Value
Participants, *n*	21	52	–
Sex, male/female	11/10	36/16	–
Age, years	28.8 ± 9.9^a^ (16–47)	25.5 ± 7.7^a^ (14–43)	–
BMI, kg/m^2^	22.6 ± 3.3^a^	25.9 ± 3.91^a^	–
Autograft type, ST/ST+G	15/6	33/19	–
Δ Tunnel diameter_1 Y–1 D_			
Femoral aperture	2.4 ± 1.1^a^	2.6 ± 1.3^a^	n.s.*
Tibial aperture	1.2 ± 0.8^a^	1.5 ± 1.0^a^	n.s.*
PROs _1Y_			
IKDC	89.4 ± 4.6^a^	89.0 ± 6.0^a^	n.s.*
Lysholm	90.0 (85.0–95.0)^b^	89.7 ± 6.5^a^	n.s.**
KOOS‐symptoms	89.3 (83.9–96.4)^b^	88.9 ± 8.1^a^	n.s.**
KOOS‐pain	94.4 (88.9–98.6)^b^	97.2 (91.7–100.0)^b^	n.s.**
KOOS‐ADL	95.6 (91.9–98.5)^b^	97.8 (95.2–100.0)^b^	n.s.**
KOOS‐sport	79.8 ± 11.0^a^	82.8 ± 13.0^a^	n.s.*
KOOS‐QoL	83.0 ± 13.4^a^	87.5 (75.0‐89.1)^b^	n.s.**
SNQ			
Distal	0.1 (−0.1 to 0.8)^b^	0.6 (0.1–1.0)^b^	n.s.**
Middle	1.9 (0.9–3.5)^b^	1.5 (0.7–2.8)^b^	n.s.**
Proximal	1.7 ± 1.3^a^	1.3 (0.8–1.8)^b^	n.s.**
Full	1.6 ± 1.0^a^	1.0 (0.8–2.2)^b^	n.s.**

*Note*: Δ Indicates the tunnel diameter difference between the first day and 1 year postoperatively.

Abbreviations: ADL, activities of daily living subscale; BMI, body mass index; G, gracilis; IKDC, The International Knee Documentation Committee; KOOS, Knee Injury and Osteoarthritis Outcome Scores; PRO, patient‐reported outcome; QoL, quality of life subscale; SNQ, signal/noise quotient; Sport, sport and recreation subscale; ST, semitendinosus; 1 D, 1st day postoperatively; 1 Y, 1 year postoperatively.

a Data are expressed as mean ± SD.

^b^ Data are expressed as median (*P*_25_–*P*_75_).

* A *p* value of <0.05 indicates statistical significance with the *t* test and is highlighted in bold.

** A *p* value of <0.05 indicates statistical significance with the Wilcoxon signed‐rank test and is highlighted in bold.

### Correlation between tunnel enlargement and clinical outcomes

Variations in aperture diameter (Δ) showed no significant correlation with PROs and SNQ values (n.s.) for both femoral and tibial tunnels (Table 8).

**Table 8:** Multiple regression analysis of confounding factors correlated with tunnel diameter difference (Δ) and SNQ value (full graft).

Independent variables	Dependent variables											
	Δ Femoral diameter_1 Y–1 D_				Δ Tibial diameter_1 Y–1 D_				SNQ_full_			
	*β*	SE	Stdβ	*p* Value	*β*	SE	Stdβ	*p* Value	*β*	SE	Stdβ	*p* Value
Age	0.01	0.02	0.05	n.s.	−0.01	0.02	−0.11	n.s.	−0.01	0.02	−0.11	n.s.
Sex	0.55	0.50	0.21	n.s.	0.26	0.39	0.13	n.s.	0.01	0.42	0.01	n.s.
BMI	0.04	0.06	0.12	n.s.	0.02	0.05	0.06	n.s.	−0.07	0.05	−0.27	n.s.
Time from surgery to follow‐up	−0.01	0.01	−0.20	n.s.	−0.01	0.01	−0.23	n.s.	−0.01	0.01	−0.29	n.s.
Meniscal injury												
Medial	−0.14	0.57	−0.05	n.s.	0.35	0.44	0.17	n.s.	−0.07	0.47	−0.03	n.s.
Lateral	−0.82	0.62	−0.27	n.s.	0.02	0.48	0.01	n.s.	0.22	0.52	0.09	n.s.
Medial + Lateral	−0.57	0.57	−0.22	n.s.	0.39	0.44	0.20	n.s.	0.46	0.48	0.22	n.s.
Chondral lesion	−0.62	0.40	−0.24	n.s.	0.07	0.31	0.04	n.s.	0.23	0.33	0.11	n.s.
Autograft	0.03	0.43	0.01	n.s.	0.16	0.33	0.09	n.s.	0.35	0.35	0.17	n.s.

Abbreviations: BMI, body mass index; SE, standard error; SNQ, signal‐to‐noise quotient; Std β, standardized coefficients; β, non‐standardized coefficients.

### Correlation between confounding factors and tunnel diameter difference and SNQ

The included confounding factors were not significantly correlated with differences in femoral and tibial tunnel diameters and SNQ values of full autografts (n.s.) (Table 8).

## DISCUSSION

The primary finding of the present study was that both femoral and tibial bone tunnels exhibited eccentrical enlargement after primary ACLR, which gradually became stable or narrowed at 1‐year follow‐up. However, widened bone tunnels did not affect clinical outcomes and graft maturity.

The incidence of ACL rupture is increasing and ACLR is widely considered as the preferable modality of treatment modality [7, 8, 15, 42]. Meanwhile, bone tunnel enlargement has become a prevalent phenomenon following primary ACLR, influenced by surgical techniques [5, 28], graft selections [1, 30] and graft fixation methods [36, 37, 38]. However, it remains unclear whether widened bone tunnels affect clinical outcomes. Therefore, it is crucial to adopt a novel measurement method with higher reliability and accuracy to assess the impact of tunnel widening on PROs and autograft healing.

2D images from plain radiographs, CT and MRI are commonly used to evaluate tunnel widening. However, these conventional methods have several limitations. Plain radiographs, presented as grey‐scale images of overlapping articular structures, have poor resolution for clearly distinguishing bone tunnels from surrounding tissues. MRI is limited in presenting bony structures and depicting tunnel walls. Additionally, it is challenging to scan exact medians or specific planes on CT and MRI, which is important for measuring the maximum diameters of bone tunnels. Given these limitations, this study adopted a visualized 3D model to measure tunnel diameters directly. In 2014, Crespo et al. [13] first constructed 3D models of bone tunnels after ACLR and provided three methods for measuring tunnel diameters: the best transverse section method, the best‐fit cylinder method and the wall thickness method. The best transverse section method showed superior reliability and validity compared to the 2D CT method. This approach was modified, segmenting the measurement planes from the articular aperture at 5‐mm intervals to comprehensively depict dynamic variations in tunnel thickness.

In this study, three general patterns of tunnel widening were observed. First, the extent of widening was greatest at the aperture level on both femoral and tibial sides. Mechanical and biological factors contributed to such enlargement [18, 46, 53]. From a biomechanical perspective, ‘windshield‐wiper’ and ‘bungee’ effects facilitated autograft micromotion in bone tunnels, likely causing tunnel enlargement, particularly at the entrance [22, 27]. In cadaveric testing, Rodeo et al. [43] indicated that extensive graft‐tunnel motion at the tunnel aperture might result in retarded graft healing and greater tunnel widening. Conversely, reducing the distance between the graft and fixation devices lessens graft‐tunnel motion and tunnel enlargement [21]. The degree of enlargement was larger in the femoral tunnel than in the tibial tunnel, consistent with several previous studies [10, 12, 38]. However, few studies have explored the underlying mechanisms of this phenomenon. We speculate that it may be due to different mechanical traction forces exerted by grafts on the femoral and tibial tunnel walls. Notably, tunnel apertures were significantly widened and then stabilized or even narrowed following primary ACLR. Previous studies have revealed that tunnel diameters mostly increase during the first 6 weeks to 6 months post‐ACLR and gradually decrease after 1 year [19, 20]. A long‐term study indicated that bone tunnels are stabilized 4 months after ACLR and narrow significantly over 15 years postoperatively [14]. Kiekara et al. [24] observed continuous tunnel enlargement up to 2 years after ACLR, followed by gradual narrowing over a 5‐year follow‐up. Additionally, Weber et al. [49] measured the cross‐sectional area of bone tunnels from 6 weeks to 2 years post‐surgery, showing that on both femoral and tibial sides, the entrance and mid‐section of bone tunnels are initially widened and then gradually decrease, while the exit consistently decrease. These findings were consistent with our study results, illustrating a dynamic change in bone tunnels after ACLR: enlargement, stabilization and narrowing. This pattern may be associated with intra‐tunnel ossification, eventually presenting as even, conical and complete narrowing, with the latter two patterns being more prevalent [24].

Positions of the tunnel apertures shifted eccentrically with tunnel widening. The autografts exerted uneven mechanical traction forces on the tunnel walls, particularly at the aperture. Thus, the bone tunnel enlarged along the direction of graft tension, resulting in enlargement and shifting of the eccentrical tunnel. Regarding the femoral tunnel, greater mechanical stress was exerted on the anterior and distal tunnel walls [23, 28, 32], and the tibial tunnels migrated along the posterior and lateral mechanical stress directions [2, 32]. In this study, the central points of femoral tunnels shifted toward anterior and distal orientations, while those of the tibial tunnels moved toward posterior and lateral directions. This has been described as eccentrical tunnel widening [32]. Additionally, both femoral and tibial tunnel shifting slowed down 1 year postoperatively, indicating a stable trend in terms of tunnel diameters.

Despite significant tunnel widening after primary ACLR, the extent of tunnel enlargement did not negatively impact knee functions, clinical scores and graft maturity. As important indicators for postoperative status, PROs were significantly improved between preoperative and 6‐month follow‐up following ACLR, which reported gradual stability or a tendency to ameliorate over time. A similar trend variation was found in previous studies [5, 16, 45, 50]. In addition, SNQ values have been widely used to assess postoperative autograft healing [6, 33, 48, 51, 52]. In this study, the distal graft exhibited superior healing capability compared with the mid‐substance and proximal parts. However, there was no evidence of a significant correlation between the degree of tunnel enlargement and PROs and SNQ values, suggesting that tunnel widening may not affect patient‐reported clinical outcomes and graft maturity after primary ACLR. In addition, these results also highlighted that more attention should be paid to patients' actual status, especially QoL and return to sports. In an earlier study, Lee et al. [29] reported that the extent of femoral tunnel widening does not affect clinical scores, whereas larger tunnel diameters contribute to inferior knee anterior stability after ACLR. Additionally, confounding factors, such as age, gender, BMI, follow‐up time, concomitant injuries and autograft type, were not significantly correlated with tunnel enlargement and graft maturity, indicating that other factors might function in important roles. In general, enlarged tunnels remain as underlying risk factors that potentially cause instability or laxity in knee joints, resulting in graft failure and posing challenges for potential revisionary surgeries.

This study had certain limitations. First, the number of enroled participants was relatively small. Therefore, we calculated the sample size based on previously published studies, and a sample size of 22 pairs was deemed sufficient. Second, the follow‐up period was relatively short. Thus, a longer follow‐up is required to depict long‐term variations in tunnel diameters and positions and investigate the potential impacts of exercise intensity on tunnel widening and graft maturity. Additionally, the 3D models of bone tunnels employed here were manually modified for a more accurate evaluation of diameters. Although this inevitably introduced subjectivity, these procedures were performed by the same experienced and independent investigator in orthopaedics. Furthermore, the latent role of graft type, fixation device and even sport intensity in relation to tunnel widening need to be further investigated.

The clinical relevance of the present work lies in controversies concerning the arising of tunnel enlargement after primary ACLR. This study found that the widened bone tunnels did not affect the quality of life of patients. Therefore, a return to normal daily life and sport was encouraged following ACLR regardless of the degree of tunnel widening.

## CONCLUSIONS

Eccentrically widened bone tunnels were gradually stabilized 1 year post‐primary ACLR, and larger tunnels were not associated with inferior clinical outcomes or graft maturity.

## AUTHOR CONTRIBUTIONS

Di Liu and Wenhao Lu collected the data, decided on the content, and wrote and revised the manuscript. Wenfeng Xiao and Yusheng Li conceptualised this article and revised the draft. Can Chen and Yusheng Li guide the rework of the manuscript and suggest revisions. Qing Bi and Zheping Hong provided guidance on manuscripts. Di Liu and Djandan Tadum Arthur Vithra made the figures. Wenhao Lu, Xu Liu and Dongliang Yuan made the tables. Wenfeng Xiao, Yusheng Li and Zheping Hong have provided fund support. All authors read and approved the final manuscript.

## CONFLICT OF INTEREST STATEMENT

The authors declare no conflict of interest.

## ETHICS STATEMENT

Ethics approval was sought and gained from the Medical Ethics Committee of Xiangya Hospital Central South University (No. 202201039). The written informed consent was obtained from all patients.

## Data Availability

The data that support the findings of this study are available on request from the corresponding author. The data are not publicly available due to privacy or ethical restrictions.
